# Development of process analytical tools for rapid monitoring of live virus vaccines in manufacturing

**DOI:** 10.1038/s41598-022-19744-x

**Published:** 2022-09-15

**Authors:** Sijia Yi, Reilly McCracken, Joseph Davide, Daniel Ryan Salovich, Travis Whitmer, Aditya Bhat, Josef Vlasak, Sha Ha, Darrell Sehlin, Joseph Califano, Kristin Ploeger, Malini Mukherjee

**Affiliations:** 1 Vaccines Process Development and Commercialization, Merck & Co., Inc., 770 Sumneytown Pike, WP 42-3, West Point, PA 19486 USA; 2 Vaccines Analytical Development, Merck & Co., Inc., West Point, PA USA; 3 Aber Instruments Inc, Alexandria, VA USA

**Keywords:** Biophysical methods, Imaging, Microscopy

## Abstract

In the development of end-to-end large-scale live virus vaccine (LVV) manufacturing, process analytical technology (PAT) tools enable timely monitoring of critical process parameters (CPP) and significantly guide process development and characterization. In a commercial setting, these very same tools can enable real time monitoring of CPPs on the shop floor and inform harvest decisions, predict peak potency, and serve as surrogates for release potency assays. Here we introduce the development of four advanced PAT tools for upstream and downstream process monitoring in LVV manufacturing. The first tool explores the application of capacitance probes for real time monitoring of viable cell density in bioreactors. The second tool utilizes high content imaging to determine optimum time of infection in a microcarrier process. The third tool uses flow virometry (or nanoscale flow cytometry) to monitor total virus particle counts across upstream and downstream process steps and establishes a robust correlation to virus potency. The fourth and final tool explores the use of nucleic acid dye staining to discriminate between “good” and “damaged” virus particles and uses this strategy to also monitor virus aggregates generated sometimes during downstream processing. Collectively, these tools provide a comprehensive monitoring toolbox and represent a significantly enhanced control strategy for the manufacturing of LVVs.

## Introduction

The race to vaccinate the world against SARS-CoV2 has led to significant efforts to compress the time to manufacture vaccines by improving control strategies around large-scale manufacturing operations. Unlike some of the newer mRNA vaccines that have well understood ways for degradation of their critical quality attributes (CQAs) and analytical assays that can reliably track and correct these changes during manufacturing^1^, LVVs have a plethora of poorly understood mechanisms by which key attributes like virus antigenicity or infectivity can be lost^2–6^. The traditional control strategy to monitor LVVs during manufacturing by measurements of cell viability and cell metabolism cannot achieve continuously monitoring cellular “fingerprints” during the viral production cycles and de-risk in a timely fashion. The assessment of the parameter optimization relies on classical cell-based potency assays, like tissue culture infectious dose 50% (TCID50) and plaque assays, which take at least 1–2 weeks and are difficult to adapt to high-throughput workflows^7^. The lack of tools to directly monitor and measure LVV potency rapidly results in longer iteration time for process development and process characterization of these products, and this is evidenced by the significantly longer times to launch LVVs against infectious diseases. PAT development to better track LVVs in real time and ensure their quantity as well as quality has thus become the focus of many big pharmaceutical manufacturers and are being increasingly recommended by regulatory agencies^8,9^.

Several methods are available for direct virus quantification, such as detection of virus proteins via enzyme-linked immunosorbent assays (ELISA), quantification of virus nucleic acid by quantitative reverse-transcription polymerase chain reaction (qRT-PCR), and both measuring viral protein or nucleic acid content by capillary electrophoresis (CE)^10^. However, these “bulk” analytical methods cannot provide direct information on single virus particles and virus aggregation^7^. As viruses are sub-visible particles in the nanoscale range, reliable tools to directly measure individual virus particles are not many. Microscopy-based techniques, such as electron microscopy (EM) and atomic force microscopy (AFM) could provide high resolution single-particle analysis for size, morphology, and concentration^11^. However, these techniques are time-consuming, labor-intensive, low-throughput and expensive, which limit their widespread utilization in LVV manufacturing^7^. Methods like Nanoparticle tracking analysis (NTA) are developed for nanoparticle size, concentration, and aggregation state characterization based on a laser-illuminated microscopy technique^12,13^ The Brownian motion of individual nanoparticles, i.e., virus particles in suspension are tracked and analyzed in real time to calculate their hydrodynamic size using the Stokes–Einstein equation. However, NTA only works when samples are suspended in a clear matrix and a relatively small concentration range is required for accurate analysis, which may be a challenge for samples with unknown concentration^7^ Flow virometry, the method described here, depends on light scattering of short wavelength (UV) light by virus particles^14^ and therefore, can only be reliably used for virions that have the size and the density to generate side scatter that can be discriminated above electronic noise. Appropriate and rigorous positive and negative controls are thus needed prior to establishing this as a tool to monitor viruses, but once established, can provide a highly quantitative and accurate measure of total virus count. Combined with protein, nucleic acid, and/or lipid dye staining, flow virometry can not only provide quantitative but also qualitative information about individual virions^15–18^.

In this report, we introduce Biocapacitance as an on-line PAT tool for continuously monitoring viral vectored vaccines production in cell culture processes. In addition, we propose high throughput potency assays for infectivity assessment of LVVs that can further speed up the process development of these lifesaving products. Moreover, we show applications of flow virometry to the near real time monitoring of two virus vaccine candidates^19,20^ and highlight their application as PAT in large scale manufacturing.

## Results

### Structures of two virus vaccine candidates

**Figure 1:**
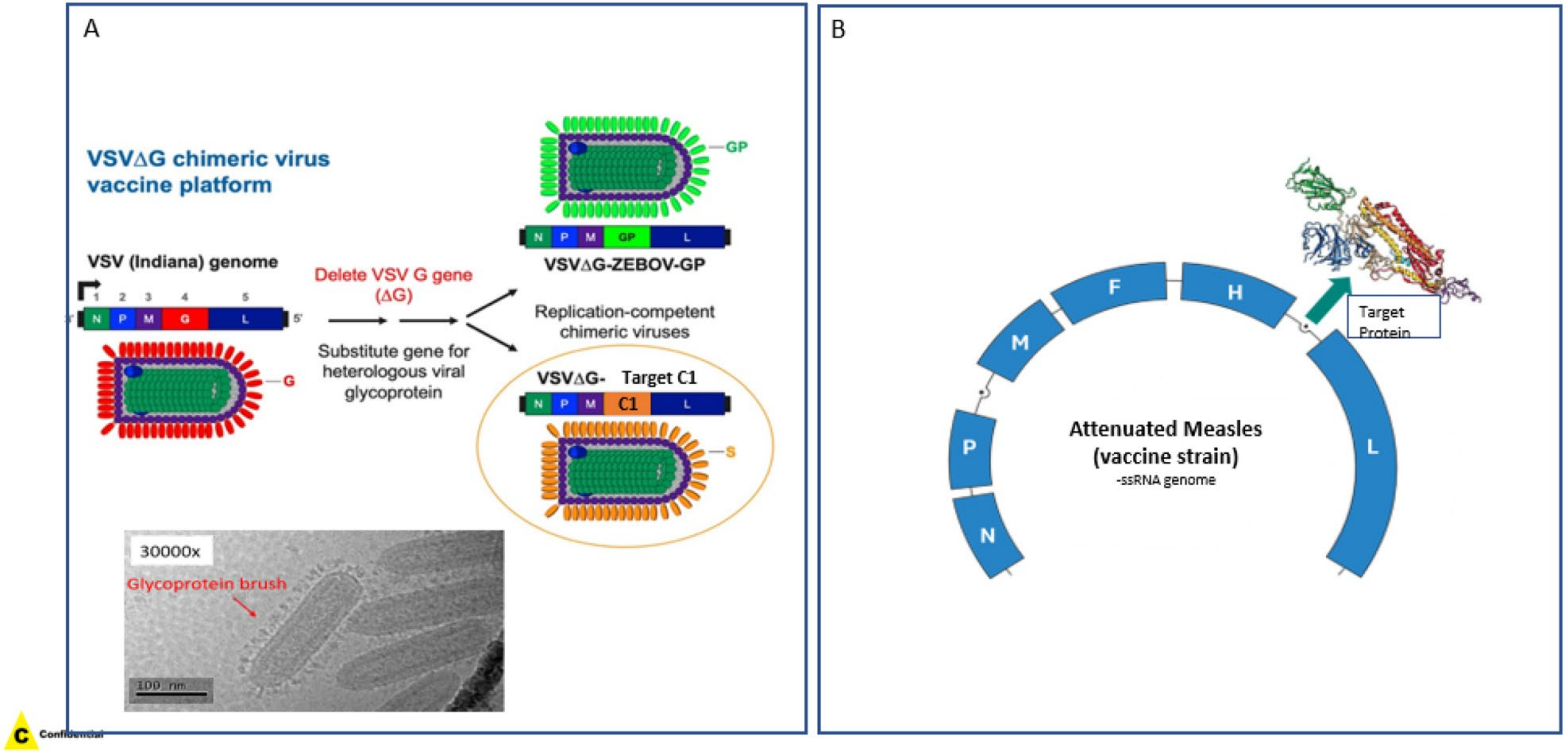
Structure of two experimental live virus vaccine candidates. (**A**) Surface glycoprotein of vesicular stomatitis virus (VSV) is replaced by target protein by genetic engineering. Cryo-EM confirms the bullet shaped structure and glycoprotein presence on surface of the VSV viral vector. (**B**) Replication competent attenuated Measles virus has a target protein sequence insert for expression upon vaccination.

Two viral vector-based vaccine candidates were designed and tested for efficacy. The first vaccine candidate (C1) used a VSV virus vector (also recently utilized to manufacture ERVEBO, our Ebola vaccine^21^, and used genetic engineering to replace VSV surface glycoprotein with the target protein (Fig. 1A). Strategy of the second vaccine candidate (C2) utilized an attenuated Measles virus as a vector to deliver the target protein sequence upon vaccination and prompt the host cells to make the target protein and subsequently generate an immune response against it (Fig. 1B). Both vaccine candidates (C1 and C2) were tested in pre-clinical studies and elicited robust immune response in primates, confirming that the delivery mechanism was successful (data not shown).

### Typical LVV manufacturing process highlights gaps in control strategy

**Figure 2:**
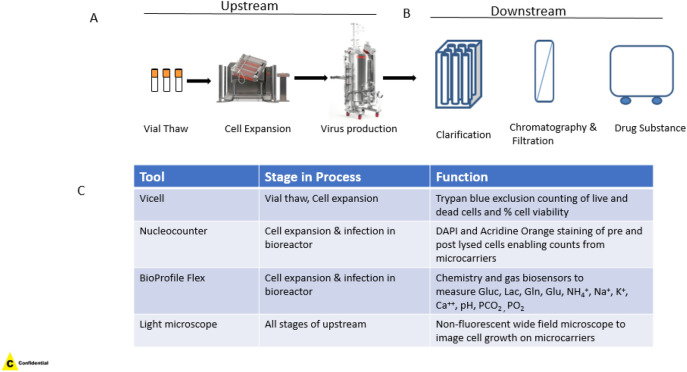
Depiction of a typical live virus manufacturing process. (**A**) Typical upstream process involves vial thaw into cell stacks that allows for optimal cell expansion after which adherent cells are planted on microcarriers to expand in a bioreactor. ViCell or Nucleocounter counts during this stage are used to monitor total and dead cell counts and cell viability. Optimally expanded cells in bioreactors are infected with virus of choice after which the production bioreactor is maintained at standard temperature and pH for virus replication. Analytical tools like Nucleocounter (NC200), BioProfile Flex and light microscopes are used to monitor cell viability, metabolites and cell expansion on microcarriers, as indirect measurements of virus growth and replication during these stages. After a fixed time of virus replication cells are harvested and virus supernatant is sent for downstream processing. (**B**) Harvested virus (HV) proceeds through a series of clarification and purification steps after which it is resuspended in formulation buffer and dispensed for freezing of final drug substance (DS). (**C**) Table of typical in-process analytical tools for live virus monitoring.

The current typical manufacturing process for LVVs can be broadly divided into upstream and downstream workflows. The upstream process generally consists of a cell growth phase, followed by viral infection and replication phase followed by the harvest of viral fluids by the addition of endonuclease to degrade cellular DNA (Fig. 2A). The harvested virus fluids (HVF) which consists of both virus particles as well as cellular debris, is then sent to the downstream process where it undergoes a series of clarification, purification, and concentration steps to remove cellular materials and enrich for virus particles (Fig. 2B). During the upstream and downstream processing, several parameters are monitored to ensure consistency of the quality and quantity of final drug substance.

Current in-process analytics embedded to exert control strategy and monitoring in manufacturing LVVs include initial cell counts and multiplicity of infection (MOI) calculations for virus seeding and subsequently bioreactor monitoring of cells for viability and metabolites (summarized in Fig. 2C). Direct measurement of virus particles is not yet a part of the routine manufacturing workflow and thus a read on virus potency is typically not obtained until generation of final drug substance upon conclusion of downstream processing. Harvest of upstream bioreactors is largely dependent on cell viability alone, leaving some significant process control gaps in confirming efficient infection of cells at the selected MOI and selecting the optimal harvest window at peak virus potency.

### Biocapacitance as a PAT for monitoring biomass and potentially C1 and C2 virus particles

**Figure 3:**
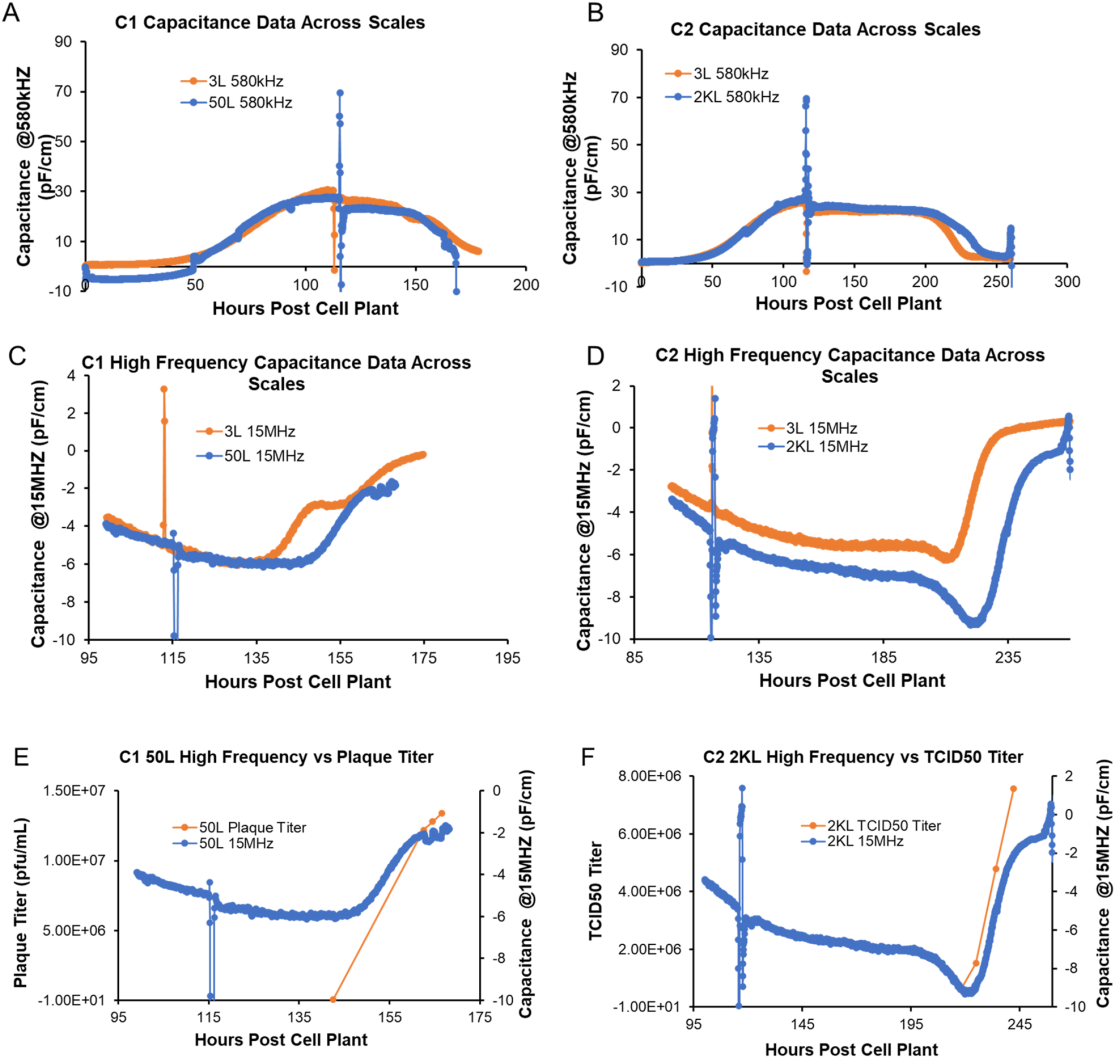
Continuous monitoring of the production process of C1 and C2 vaccine candidates. (**A**) The capacitance profile of C1 vaccine at 580 kHz in a 50L bioreactor (blue) and a 3L bioreactor (orange) in hours post cell plant into the reactors. (**B**) The capacitance profile of C2 vaccine at 580 kHz in a 2000L bioreactor (blue) and a 3L bioreactor (orange) in hours of runtime. (**C**) The capacitance profile of C1 vaccine at 15 MHz in a 50L bioreactor (blue) and a 3L bioreactor (orange) in hours post cell plant into the reactors. (**D**) The capacitance profile of C2 vaccine at 15 MHz in a 2000L bioreactor (blue) and a 3L bioreactor (orange) in hours of runtime. (**E**) The capacitance profile of C1 vaccine at 15 MHz in a 50L bioreactor (blue) and corresponding plaque assay infectivity data (orange). (**F**) The capacitance profile of C2 vaccine at 15 MHz in a 2000L bioreactor (blue) and corresponding TCID50 infectivity data (orange).

Biocapacitance probes work by using an alternating electric field to polarize live cells and the buildup of charge around live or intact cell membranes contribute to capacitance. Unlike the live cells, dead cells without intact membrane cannot store charges inside them. Since the resulting capacitance measurement is proportional to the total cellular membrane bound volume (Ref. Aber website: https://aberinstruments.com/biotech/capacitance-measurement/), biocapacitance has been used for real-time monitoring of biomass or biovolume in cell culture processes^22,23^. For both the C1 and C2 viral vector processes, the capacitance measured at a frequency of 580 kHz models the cellular state in a scale independent manner (Fig. 3A, B). In terms of monitoring biomass, we observed all the hallmarks of the prototypical growth curve as measured by orthogonal tools like the Nucleocounter (data not shown). At the start of the process, we observed lag phase in the first 50 h, followed by exponential growth in the next 50 h (50–100-h period). Around the 110–120-h mark during the growth plateau (stationary phase) a cell culture media exchange was performed. Compared to no media exchange condition, during media exchange, agitation was stopped and microcarriers along with cells were away from the measurement field of biocapacitance probe which resulted in a sharp vertical line indicating temporary loss of measurement (Fig. 3A, B, Figure S1). Media exchange was followed by infection where the live cells then begin their decline (death phase) at a rate which is specific to kinetics of the infectious virus particle, leading to a time-dependent decrease in capacitance at 580 kHz. In addition to monitoring biomass, we observed a novel use case to potentially monitor virus directly in real time. It is believed that virus is too small to be directly measured by the probe. However, since both these viral vectors are enveloped viruses, they theoretically can be polarized and thus be registered by the probe. If virus detection is possible, it would register at the higher frequencies due to their ability to detect smaller particles. This is exactly what we observed for both viral vectors , whereby capacitance at 15 MHz increased after infection (Figs. 3C, D), while capacitance at 580 kHz decreased due to host cell death (Fig. 3A, B). Furthermore, the increase in the high frequency (15 MHz) capacitance appears to also correlate with increases in viral titer (Fig. 3E, F, Figure S2). These observations could merely be coincidental due to the timing of the process and events like post infection biomass decline/cell death (Fig. 3A, B) due to virus replication and production. To rule this out, we carried out mock infections to examine changes in the high frequency capacitance data (Figure S3). We observed no late stage increase in high frequency capacitance without virus being present (N = 3). Therefore, we hypothesize that host cells and enveloped virus could be captured by two different frequencies of capacitance independently due to the difference in size. All results suggested that multi-frequency capacitance would not only allow the on-line monitoring of cell production kinetics, but also be employed for prediction of the optimal time of harvest.

### High content imaging identifies optimum time of infection and media exchange condition for virus infection

We have previously established the utility of high content imaging as a robust tool for potency measurements with a faster turnaround time than traditional potency assays^17^. Here we establish a way to use the same tool to optimize time of infection (TOI) based on optimal cell density on each individual microcarrier and media exchange parameters for LVV programs using microcarrier cultures. Briefly, microcarrier cultures that had grown for 4 or 5 days (4- or 5-days post plant (DPP)) were infected with C1, and cells were fixed directly on the microcarriers 24 h post infection. In addition, 5DPP cells infected with C1 were also compared between bioreactors that had undergone a media exchange (MX) or had not had a media replacement (NMX) prior to infection. Fixed samples were stained with a primary anti-Target protein (expressed on surface of C1) specific monoclonal antibody conjugated to Alexa Fluor 488, anti-VSV primary antibody with Alexa Fluor 568 secondary antibody, and Hoechst 33342 nucleic acid dye. We then used automated high content imaging to analyze ~ 100 microcarriers per condition.

**Figure 4:**
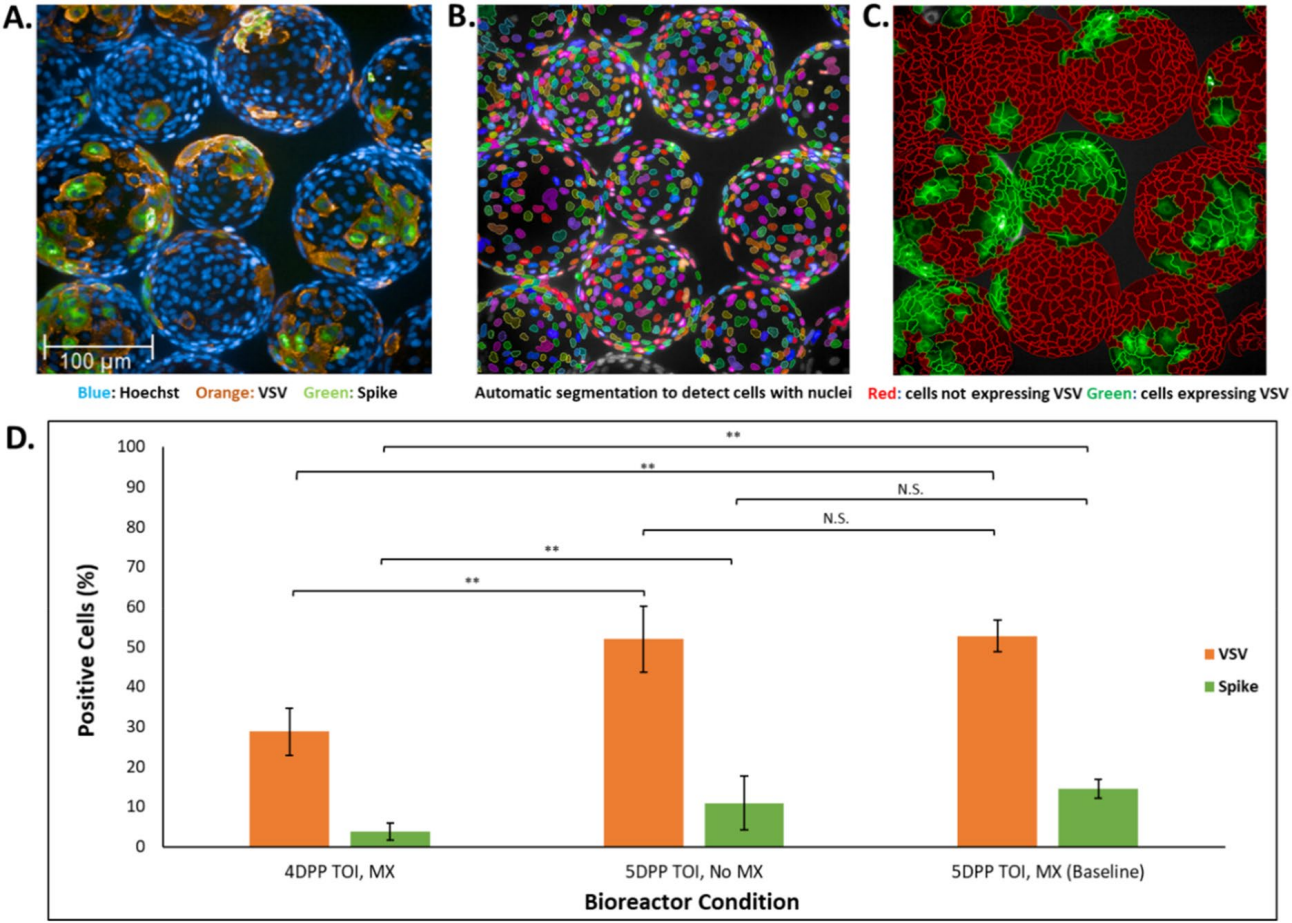
Quantitative high content imaging assay identifies optimal TOI and media condition for virus infection on microcarriers. (**A**) Image of adherent Vero cells on microcarriers stained with anti-VSV, anti-Target protein antibodies (orange and green, respectively) and Hoechst 33342 nucleic acid stain (blue). Image shown is of a single field of view in a single imaging well, taken with 20× water objective on Operetta CLS high content imager. (**B**) Segmented nuclei as identified by Perkin Elmer Harmony software algorithm, using Hoechst 33342 as a guide. Each colorful shape represents a single nucleus from which the software can segment individual cells. (**C**) Results from quantitative analysis performed on Operetta using Harmony software to distinguish cells positive for VSV or Target protein (green) and uninfected cells (red). (**D**) Comparison of the percentage of cells positive for VSV (orange) or Target protein (green) expression plotted for each bioreactor condition tested on X- axis (N = 9). **Indicates statistical significance between conditions (*p* < 0.01 for two-tailed Student’s t-test). N.S. indicates no statistical significance between conditions.

We detected both VSV and target proteins for all conditions studied, as evidenced by orange and green fluorescent expression, respectively (Fig. 4A). Using image analysis software, we determined the percentage of cells expressing VSV and Target proteins (Fig. 4B, C) for each condition. We found that the percentage of cells expressing VSV and Target protein was greater at the 5-day post plant (DPP) TOI condition compared to 4DPP (Fig. 4D). By contrast, for samples infected on 5DPP, we found no significant difference in the percentage of cells expressing VSV or Target proteins for bioreactors where no MX was performed compared to the baseline condition (Fig. 4D).

### Flow virometry acts as a process monitoring tool for both vaccine candidates

We have previously established that flow virometry can be used to directly measure and count virus particles and can thus, be effectively utilized as an at-line process monitoring tool for LVV manufacturing^17^. To evaluate if this tool can be used for the two new virus-vectored vaccine candidates, we first assessed if C1 and C2 can be detected using violet laser side scatter (VSSC).

**Figure 5:**
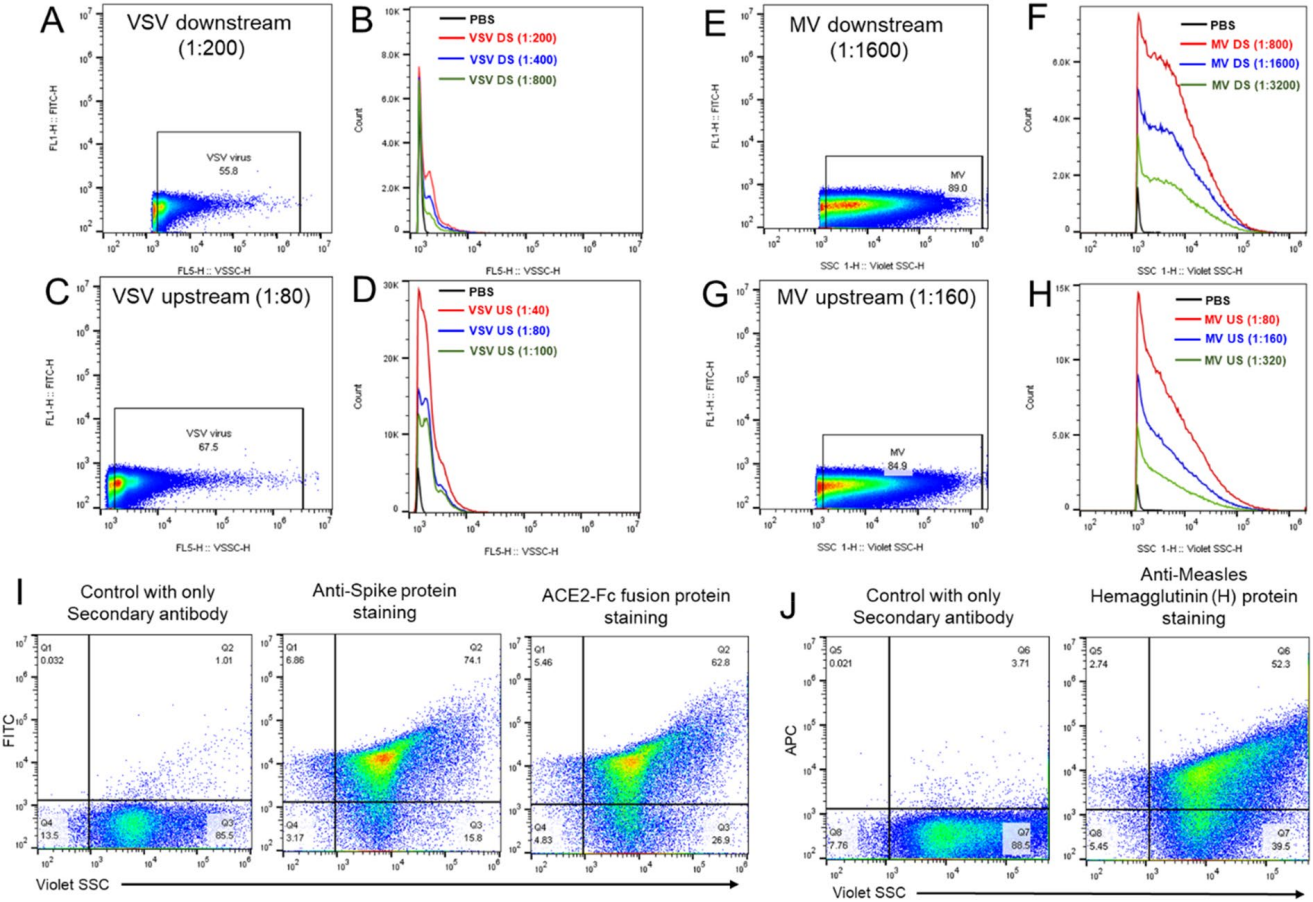
Violet laser side scattering (VSSC) profiles and antibody staining confirmed detection of individual virus particles by flow virometry for C1 and C2 vaccine candidates. (**A**) Dot plot of a representative C1 vaccine downstream drug substance was analyzed by CytoFLEX. C1 viral particles were detected as a monodisperse population that could be resolved by VSSC alone. Gating strategy was set based on VSSC of PBS background. Numbers in the gate indicated the percentage of particles in total events. (**B**) CytoFLEX histogram overlays of PBS background and C1 vaccine downstream drug substance in 0.1 µm filtered PBS buffer at a dilution of 1:200, 1:400, and 1:800. Single peaks from C1 viral particles were detected at optimal dilutions for the purified downstream sample. (**C**) Dot plot of a representative C1 vaccine upstream sample harvested from bioreactors was analyzed by CytoFLEX. (**D**) CytoFLEX histogram overlays of C1 vaccine upstream harvest sample in 0.1 µm filtered PBS buffer at a dilution of 1:40, 1:80, and 1:100. Peaks with right shoulders were observed at optimal dilutions for bioreactor samples, suggesting the cellular components or impurities available in upstream samples. (**E**) Dot plot of a representative C2 virus vaccine downstream drug substance was analyzed by CytoFLEX. Widely distributed populations were detected for C2 virus. Gating strategy was set based on VSSC of PBS background. Numbers in the gate indicated the percentage of particles in total events. (**F**) CytoFLEX histogram overlays of C2 vaccine downstream drug substance in 0.1 µm filtered PBS buffer at a dilution of 1:800, 1:1600, and 1:3200. Broad peaks were detected at optimal dilutions for the purified downstream sample. (**G**) Dot plot of a representative C2 vaccine upstream sample harvested from bioreactors was analyzed by CytoFLEX. (**H**) CytoFLEX histogram overlays of C2 vaccine upstream harvest sample in 0.1 µm filtered PBS buffer at a dilution of 1:80, 1:160, and 1:320. (**I**) Representative plots of C1 vaccine stained with anti-Target protein antibody or cellular receptor fusion protein antibody followed by a secondary antibody conjugated with AF488 fluorescence. Violet laser side scattering (VSSC) defines virus population on X-axis, while FITC fluorescent signal on Y-axis indicates membrane staining with anti-virus target protein antibody or cellular receptor fusion protein antibody. Gating strategy was set based on viral particles stained without primary antibodies but with only secondary antibody conjugated with AF488. The upper right quadrant (Q2) of each plot represents virus surface protein-positive virus particles. (**J**)**.** Representative plots of C2 vaccine stained with anti-C2 surface protein antibody followed by a secondary antibody conjugated with AF647 fluorescence. Violet laser side scattering (VSSC) defines virus population on X-axis, while APC fluorescent signal on Y-axis indicates membrane staining with anti-Measles H protein antibody. Gating strategy was set based on Measles virus stained without the primary antibody but with only secondary antibody conjugated with AF647. The upper right quadrant (Q2) of each plot represents C2 surface protein-positive virus particles.

For C1 (Fig. 5A, B), we found that a distinct population of the virus could be detected and separated clearly from the noise signal in scatter plot and was demarcated by appearance of a single narrow peak in histogram (Fig. 5B) in highly purified downstream samples. Similar distinct population enumerated by a single peak and a small right shoulder were found for C1 upstream samples (Fig. 5C, D). For C2, the VSSC profile was different from C1, in that a single defined peak was not visible, but rather a broad peak was detected for both upstream and downstream samples (Fig. 5E–H). We attributed this to the well-established heterogeneity in particle size in Measles virus^24^. To further confirm identity of the viruses being detected by flow virometry, we performed antibody staining. For C1, we stained samples with anti-virus surface protein specific monoclonal antibody and the viral entry receptor specific fusion protein, using secondary single stained samples as controls (Fig. 5I). The positive diagonal shift in the Y-axis for virus particles stained with surface anti-Target protein confirmed the identity of the virus by this method. Further confirmation was done by using fluorescently labelled virus receptor protein to capture virus particles prior to visualizing them on the flow cytometer. We reasoned that previously identified virus surface (target) protein on the virions would bind robustly to the known cellular receptor fusion protein resulting in a near complete shift in the VSSC virus population towards the Y-axis. This shift confirmed that this method was in fact detecting virus particles. For C2, we stained the bioreactor and downstream concentrated samples with two different and specific anti-C2 surface protein antibodies, both of which are expected to be expressed at varying levels on the surface of the C2 virions. We saw a large population of positively stained viruses on the Y-axis (Fig. 5J) confirming the surface identity of these particles.

### Flow virometry as a surrogate for potency for Upstream and Downstream process development of C1

**Figure 6:**
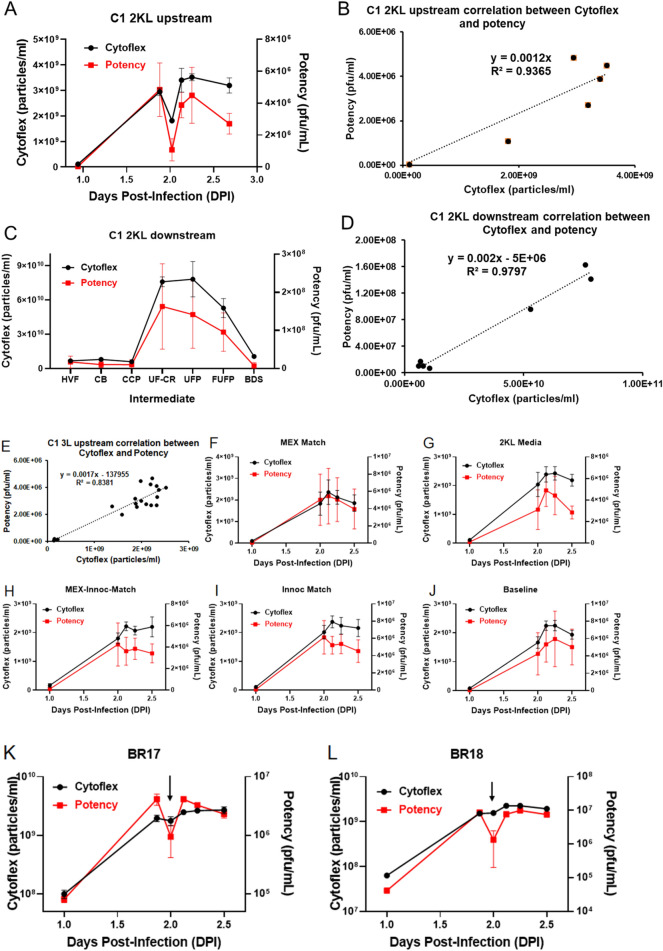
The production of C1 manufactured in 3L and 2000L bioreactors was monitored by CytoFLEX and showed strong correlation with potency. (**A**) Potency and total particle count of C1 in 2000L bioreactor samples at different days post infection (DPI). (**B**) Correlation between total particle count measured by CytoFLEX and potency obtained from Micro-plaque assay for C1 in 2000L bioreactor samples (R = 0.89, N = 6). (**C**) Potency and total particle count of C1 in 2000L after each downstream process step for virus harvest, clarification, endonuclease reaction, purification, and concentration: harvested virus fluid (HV), clarified bulk (CB), capto core product (CCP), ultrafiltration concentration (UF-CR), ultrafiltered product (UFP), final ultrafiltered product (FUFP), and bulk drug substance (BDS). (**D**) Correlation between total particle count measured by CytoFLEX and potency obtained from Micro-plaque assay for C1 2000L downstream intermediates (R = 0.99, N = 7). (**E**) Correlation between total particle count measured by CytoFLEX and potency obtained from Micro-plaque assay for C1 3L bioreactor samples (R = 0.92, N = 20). The trends of total particle count monitored by CytoFLEX and potency tested by Micro-plaque assay over time in 3L bioreactors under different conditions: (**F**) MEX Match: separate media formulations where media were formulated in two independent locations. (**G**) 2KL Media: media formulated for 2000L bioreactor and transferred to the 3L reactor. (**H**) MEX-Inoculation-Match: media exchange to match cell-adhered microcarrier settling time. (**I**) Inoculation Match: cell hold time between n-1 harvest and production bioreactor plant matching 2000L cell transfer time. (**J**) Baseline: 3L scale down model control. (**K**, **L**) The yield and titer of C1 3L bioreactors with transient issues were tested by CytoFLEX and Micro-plaque assay.

Flow virometry provides fast measurements of total particles including both infectious virions and non-infectious particles, while cell-based potency assays quantify only infectious virus particles. The ratio of total-to-infectious particles ranges widely from ten to several million for different viruses due to the presence of defective damaged or empty viral particles that do not contribute to an infectious titer^25,26^. To generate correlations to potency and evaluate flow virometry as a surrogate for potency monitoring, we first trended total virus particle counts for upstream bioreactors from 2000L scales and compared these to plaque potency results. Directionally, the trends for total particle count and potency matched closely and provided a robust correlation (Fig. 6A, B). The same was observed for a scale down model (3L, Fig. 6E). Next, to pressure test this method and understand if subtle changes in bioreactor conditions were reflected in changes in total particle counts, we tested a variety of conditions in the 3L scale. The conditions tested and compared to baseline 3L bioreactor were: (1) Separate media formulations where media was formulated in two independent locations (Fig. 6F), (2). Media formulated for 2000L bioreactor and transferred to the 3L reactor (Fig. 6G), (3) Media exchange to match cell-adhered microcarrier settling time (Fig. 6H), (4) Cell hold time between n-1 harvest and production bioreactor plant matching 2000L cell transfer time (Fig. 6I) and (5) 3L scale down model control (Fig. 6J). For each of these conditions, identical trends for total particle count and potency were observed. In addition, a comparison between the total particle count using either CytoFLEX or Apogee shows particularly good agreement between the two types of flow virometry (Figure S5). Interestingly, some bioreactors showed a sharp dip in total particle count and recovery hereafter, also reflected by plaque potency (Fig. 6K, L, arrow). It indicated that CytoFLEX could rapidly monitor the changes, which might not be detected in current control strategies, in the virus production process.

Like upstream we also monitored total virus particle counts across all intermediates of downstream purification. We found the trends and directionality to match plaque potency for all intermediates (Fig. 6C) across scales (3L and 2000L). Statistical analysis of correlation between total particle and plaque potency showed robust predictive value of one using the other (Fig. 6D).

### Flow virometry as a PAT for upstream and downstream process development for C2

**Figure 7:**
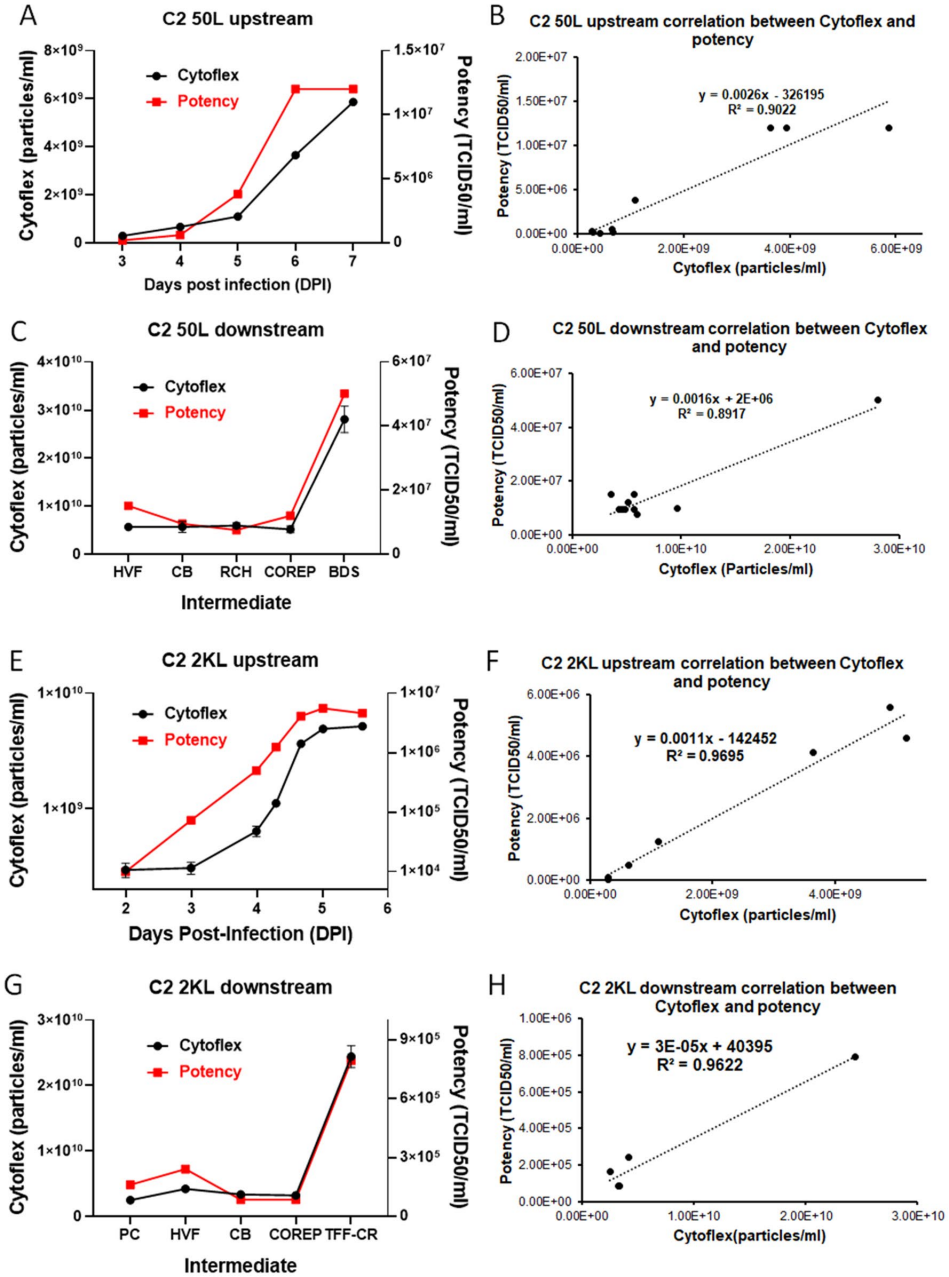
The production of C2 manufactured in 50L and 2000L bioreactors was monitored by CytoFLEX and showed strong correlation with potency. (**A**) Potency and total particle count of C2 50L bioreactor samples at different days post infection (DPI). (**B**) Correlation between total particle count measured by CytoFLEX and potency obtained from TCID50 assay for C2 50L bioreactor samples (R = 0.95, N = 9). (**C**) Potency and total particle count of C2 50L after each downstream process step for virus harvest, clarification, endonuclease reaction, purification, and concentration: production culture (PC), harvested viral fluid (HVF), clarified bulk (CB), capto core product (COREP), and tangential flow filtration concentrated retentate (TFF-CR). (**D**) Correlation between total particle count measured by CytoFLEX and potency obtained from TCID50 assay for C2 50L downstream intermediates (R = 0.94, N = 10). (**E**) Potency and total particle count of C2 2000L bioreactor samples at different days post infection (DPI). (**F**) Correlation between total particle count measured by CytoFLEX and potency obtained from TCID50 assay for C2 2000L bioreactor samples (R = 0.98, N = 7). (**G**) Potency and total particle count of C2 2000L intermediates during downstream purification. (**H**) Correlation between total particle count measured by CytoFLEX and potency obtained from TCID50 assay for C2 2000L downstream intermediates (R = 0.98, N = 5).

Like the VSV vaccine platform, we evaluated the application of flow virometry to the Measles platform (C2). Data from upstream 50L and 2000L bioreactors was collected for the full length of production time and plotted for total particles and TCID 50 potency (Fig. 7A–D). Once again, we saw similar and near identical trends in both and a robust correlation (R > 0.9). For downstream samples, total particle counts for all intermediates in purification were plotted alongside TCID 50 potency values (Fig. 7E–H). The intermediate yield determined by CytoFLEX showed similar trends and a strong correlation (R square > 0.95) with TCID 50 potency data. Moreover, two types of flow virometry (CytoFLEX and Apogee) showed identical results for both upstream and downstream viral particle measurements (Figure S4, S5). All these results indicate that CytoFLEX could be used as a PAT tool for rapid measurement of Measles virus yield for both upstream and downstream process monitoring.

### Flow virometry as a PAT for monitoring virus integrity in LVV manufacturing

**Figure 8:**
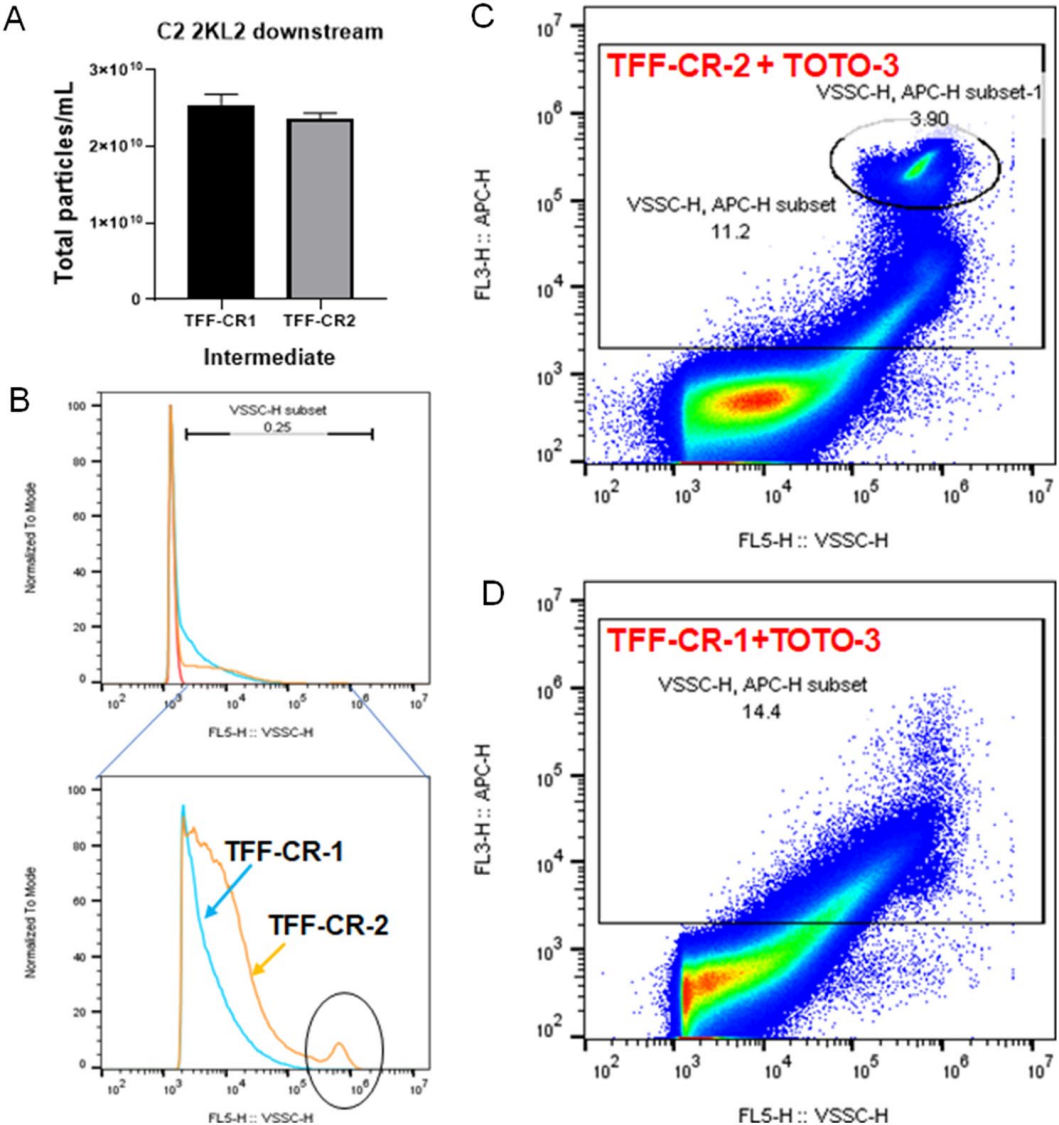
The integrity of C2 virus particles was rapidly detected using CytoFLEX during 2000L downstream purification process. (**A**) Total particle counts of C2 2KL downstream samples TFF-CR1 and TFF-CR2 were measured by CytoFLEX. (**B**) Histogram overlays of PBS, TFF-CR1, and TFF-CR2 were measured by CytoFLEX. The zoomed-in histogram plot showed different light scattering profiles between TFF-CR1 and TFF-CR2. The circled peak indicated the larger sized “aggregates” from TFF-CR2. (**C**, **D**) Dot plots of violet laser side scatter (X-axis) versus TOTO-3-APC (Y-axis) were generated from TFF-CR2 (**C**) and TFF-CR1 (**D**) stained with an impermeable nucleic acid dye TOTO-3. The circled population with TOTO-3-APC positive particles in (**C**) suggested the presence of aggregated damaged/dead virions formed in TFF-CR2 samples.

We have established before the application of flow virometry in monitoring not just particle quantity but also quality using a VSV viral vector^17^. Here we wanted to explore a similar application in monitoring virus quality using the C2 virus vaccine. As part of process development, we had generated two downstream intermediate samples, both of which had undergone identical upstream production and downstream clarification steps. The only differences in generation of the two intermediates was an increased flow rate (39 L/min vs 19.5 L/min) and virus sample concentration factor being targeted (26.8 × vs 14.5×) during tangential flow filtration (TFF), which resulted in increased shear stress. The sample generated at 19.5 L/min had a TCID50 potency value within expected range, but the sample generated at 39 L/min had no detectable potency using the same assay. Total particle counts measured by CytoFLEX were not significantly different between the two samples (Fig. 8A). We hypothesized that compromised virus quality like virus membrane damage caused by increase in shear stress created by higher flow rate could result in loss in potency and a decrease in total particle counts due to aggregation. To test this hypothesis, we stained both samples with a membrane impermeable nucleic acid staining dye (TOTO-3). We found a distinct and strong population of TOTO-3 positively stained population in the samples generated at 39 L/min (Fig. 8C), but a background “smear” signal of TOTO-3 positive population in the samples generated at 19.5 L/min (Fig. 8D). Back gating of this distinct population to the virus histogram from violet laser side scattered confirmed these to be in the small but visible right shoulder that appeared in the unstained 39 L/min sample but was not present in the 19.5 L/min sample (Fig. 8B). This test indicated that a distinct population was created in the virus samples generated with higher pressure, that had damaged membranes and were not contributing to infectious virus particles, thus resulting in a significant loss of potency.

## Methods

### Cells

Vero cells used in this study were obtained from ATCC (CCL-81.2). Cells were cultured in OptiPRO™ Serum Free medium (Thermo Fisher Scientific) supplemented with 4 mM L-glutamine (Thermo Fisher Scientific) and propagated in a humidified incubator (Thermo Fisher Scientific) at 37 °C with 5% CO_2_.

### Viruses

The vesicular stomatitis virus (VSV) vectored virus and Measles vectored virus used in this study were obtained from MSD laboratories.

### Apogee and CytoFLEX assays

The Apogee A60 is configured with 405 nm violet laser and forward, side, and green fluorescence detectors. Apogee refers to traditional forward and side scatter as small-angle light scatter (SALS) and large-angle light scatter (LALS) respectively. The CytoFLEX S is in standard configuration with three color lasers and assorted detectors; for the purposes of this study, we are concerned primarily with violet side scatter (VSSC, analogous to the Apogee 405 nm LALS). No customization was required of the equipment after initial set up.

Highly filtered sheath fluid: either 0.1 µm phosphate buffered saline (PBS), 0.12 µm Milli-Q water, or purchased Beckman Coulter sheath fluid, were used for all of these studies. Both cytometers were confirmed to be capable of visualizing and enumerating control beads and viral particles with control samples prior to using for experimental conditions**.**

All samples were diluted in 0.1 µm filtered PBS and run on Apogee and CytoFLEX Flow Virometers, dilutions are noted in the sample names on Figures. For Apogee, a flow rate of 1.5 µL/min was selected and for CytoFLEX a flow rate of 10 µL/min was used. Total particles count for samples was measured by multiplying events/second recorded with dilution of the sample and total volume collected during each run. The profile of each sample was recorded and analyzed using FlowJo software.

Control polystyrene beads for confirming operation of flow cytometers were sourced through Apogee and utilized per manufacturer recommendations. The Apogee A60 utilizes a traditional photomultiplier tube (PMT) with variable voltage, settings were identical for all profiles which we compared to one another.

For antibody staining of virus particles, samples were directly stained with primary and secondary antibodies of choice in PBS and purified between staining to remove unbound antibody using a Capto Core 700 column. Stained samples were detected on the Y-axis of the Apogee and CytoFLEX flow cytometers using the relevant fluorescence detection channel.

### High content imaging

Uninfected and C1 infected Vero cells on microcarriers were sampled from 3L bioreactors of varying experimental conditions at 4 h pre-infection and 24 h post infection, respectively. Samples were fixed and permeabilized using BD Biosciences Cytofix/Cytoperm™ kit and blocked with 4% goat serum. Infected cells were stained sequentially with anti-VSV primary antibody, Alexa Fluor 568 secondary antibody, anti-Spike primary antibody conjugated to Alexa Fluor 488, and Hoechst 33342 nuclear dye in 1X BD Perm/Wash™ Buffer. Uninfected cells were stained using the same sequence to act as negative controls. Secondary antibody controls were prepared by staining infected cells with Alexa Fluor 568 or Alexa Fluor 488 and Hoechst 33342 only. After staining, samples were transferred to a Cell Carrier Ultra 96-well Microplate (Perkin Elmer). High content imaging was performed using the 20× water objective on the Operetta CLS™ in confocal mode with Digital Phase Contrast, Hoechst 33342, Alexa 568 and Alexa 488 imaging channels. ~ 20,000–30,000 cells (~ 150–250 microcarriers) were analyzed in each condition. Cells were determined to be “positive” for VSV or Target protein expression if their mean A568 or A488 fluorescent intensity was greater than 500 based on the fluorescent intensities of unstained, secondary alone and uninfected controls. Cells with a mean A568 or A488 fluorescent intensity less than 500 were determined to be “negative” for VSV or Target protein expression.

### Biocapacitance

Biocapacitance data was collected at multiple frequencies from Aber 220 mm Annular probes (Aber:Futura Probes:6532-52/PG) using the provided software (FuturaTool V3.0.5.0) in accordance with the manufacturer’s recommended setup instructions. Briefly, the probe was connected to the head amplifier (Aber:Futura Hardware:2330-00) which was connected to the data hub (Aber:Futura Hardware:2801-00). Data hub was connected to a laptop running the Aber software (FuturaTool V3.0.5.0) via USB. Multiple frequency data was outputted as a CSV file with post run analysis in Microsoft Excel (version: Microsoft 365 Apps for enterprise).

### Data analysis

Operetta CLS high content imaging data was analyzed using Perkin Elmer Harmony 4.9 analysis software. Each experimental condition includes data from three unique 3L bioreactors tested in triplicate (N = 9) and are expressed as means ± standard deviation. Conditions were compared using a two-tailed Student’s *t*-test and a *p* value < 0.05 was considered statistically significant. Microsoft Excel was used to generate graphs and perform statistical analysis.

Flow cytometry data was analyzed using Flowjo 10.6.2. Graphs were generated using GraphPad Prism 8 (GraphPad Software). The correlation was analyzed by linear regression between CytoFLEX total particle count and potency. Pearson’s correlation coefficient and the coefficient of determination (R2) were analyzed with Microsoft Excel and GraphPad Prism 8.

## Discussion

The large-scale commercial manufacturing of LVVs consists of a series of steps starting from the culture and expansion of mammalian cells, followed by infection with virus of interest and the subsequent purification of the viral material in large quantities to finally be vialed and distributed. Throughout this process, a series of analytical measurements are implemented to ensure the quantity and quality of the final product and exert suitable control over the manufacturing process. Such controls notwithstanding, the risk of a low potency investigation in LVV manufacturing remains a pertinent risk and a challenge for pharma companies. The need for more real time monitoring of biopharmaceutical processes using PAT tools has been discussed by regulatory agencies in recent years and is increasingly becoming a regulatory expectation (ICH guidelines). The Covid-19 pandemic has further highlighted the need for efficient and fast large-scale manufacturing of vaccines with a quick turnaround time and minimal waste, which most traditional LVV processes are not yet equipped for. A typical LVV process can take years of process development and many iterations of troubleshooting to establish the final method. One of the bottle necks for process development work in the LVV space is the long turnaround time for potency assays. While several unified and platform technologies are being used for the commercial upstream and downstream manufacturing of vaccines such as the use of Vero and CHO cell lines, such a unified approach to the application of PAT tools is still missing. Here we showcase a set of PAT tools that can be effectively used for process monitoring and potency prediction of many different LVVs. Advancing prior work on application of PAT tools to support investigations around low potency^27^, here we look at a more mainstream application of these tools on two different virus vectors and show that both can be quantitatively and qualitatively monitored using these methods.

Measurement of cell viability and monitoring of the expected initial increase followed by the post infection decline is one of the most critical process parameters for LVV manufacturing. Most established methods are “at line” which means they require manual sampling and tend to be labor intensive and error prone. With the application of capacitance probes to measure biomass of adherent cells growing on microcarrier cultures in real time, we were able to advance monitoring of cell viability significantly for LVV cultures, whereby, past applications of capacitance probes have mostly been limited to applications in suspension mammalian and microbial cell cultures^22,23,28–33^. Our model confirms that in-line real-time monitoring by probes can be used to replace the more traditional ways to measure cell viability. In addition, we present here the intriguing possibility of directly monitoring virus particle biomass using capacitance at a higher frequency, which, if confirmed by more rigorous future studies, would present a PAT tool that could offer a way to obtain potency results in real time and would result in significantly enhanced process control and time saving.

Virus particle counting by flow virometry is a biophysical measurement of the virus and does not directly measure virus infectivity. However, unlike true potency measurements, these methods are usually easier and significantly faster. To use this tool as a proxy for potency though, first an optimal volume of data needs to be generated under a relevant range of manufacturing conditions and design of experiment (DOE) studies for parameters like time, temperature, pH, MOI and cell density to establish the correlation. The assumption in using such a measure as a surrogate method for potency is that for a consistent and repeatable process, the total to infectious particle ratio will remain constant. Conversely, any changes in total particle counts as measured by these means will raise concerns around virus potency and will be a suitable in-process red-flag meant to offset large scale batch failures due to OOS potencies. In addition to generating a critical mass of data, direct antibody staining studies are essential to confirm identify of particles being detected by these methods and establish that these can be discriminated from cellular vesicles of similar size. As it has been shown by others, bioreactors can also produce non-viral particles of similar size which could be a mixture of extracellular vesicles (EVs), exosomes, etc. Thus, it is not enough to confirm virus identity by staining with an extracellular antibody that can also be present in EVs, but one must also try to confirm the presence of an intra-viral protein to ensure that the particles being tracked by this method is largely the virus of interest. It is expected and not unusual that some non-viral particles as well as cellular components will be retained in this mix, especially in upstream processes, and are gradually removed upon downstream purification. Here we confirm identity of the particles using virus specific antibodies as well as rely on strong correlation to established potency assay prior to utilizing these tools for routine process monitoring. Once established, such confirmatory staining no longer needs to be performed to prevent these methods from becoming long and tedious.

Finally, high content imaging offers an attractive PAT for at-line monitoring of cells and the off-line measurement of potency. With advanced image analysis software that can measure multiple parameters within seconds and the potential to visualize them in centralized data repositories like SIPAT and Delta V, such advanced applications are now well within the reach of routine manufacturing.

## Supplementary Information

Supplementary Figures.

## Data Availability

The data that support the findings of this study are available from Merck Manufacturing Division (MMD) labs but restrictions apply to the availability of these data, due to the proprietary nature of data which were used that complies with the requirements of the current legal framework at Merck, and so are not publicly available. Data pseudo-anonymized are however available from the MMD labs upon reasonable request to any researcher wishing to use them for non-commercial purposes and will have to be approved by Merck legal prior to sharing. Researchers who wish to obtain a copy of the data may submit their request to mukhemal@merck.com.
