# Supplementary material for: Development of process analytical tools for rapid monitoring of live virus vaccines in manufacturing
Source: Sci Rep. 2022 Sep 15;12:15494. doi: 10.1038/s41598-022-19744-x (PMC9476422; doi:10.1038/s41598-022-19744-x)
Supplement: Supplementary file 1 — Supplementary Figures. [file 41598_2022_19744_MOESM1_ESM.pdf]

Supplementary information

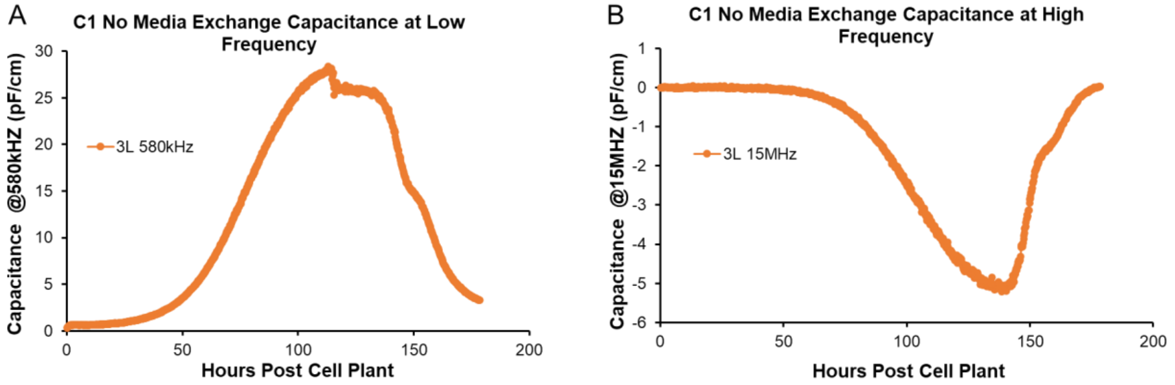

Figure S1. Biomass time profile of VSV-vectored vaccine (C1) in a 3L bioreactor without the media exchange unit operation preformed in hours post cell plant measured at **(A)** 580kHz. and **(B)** high frequency (15MHz).

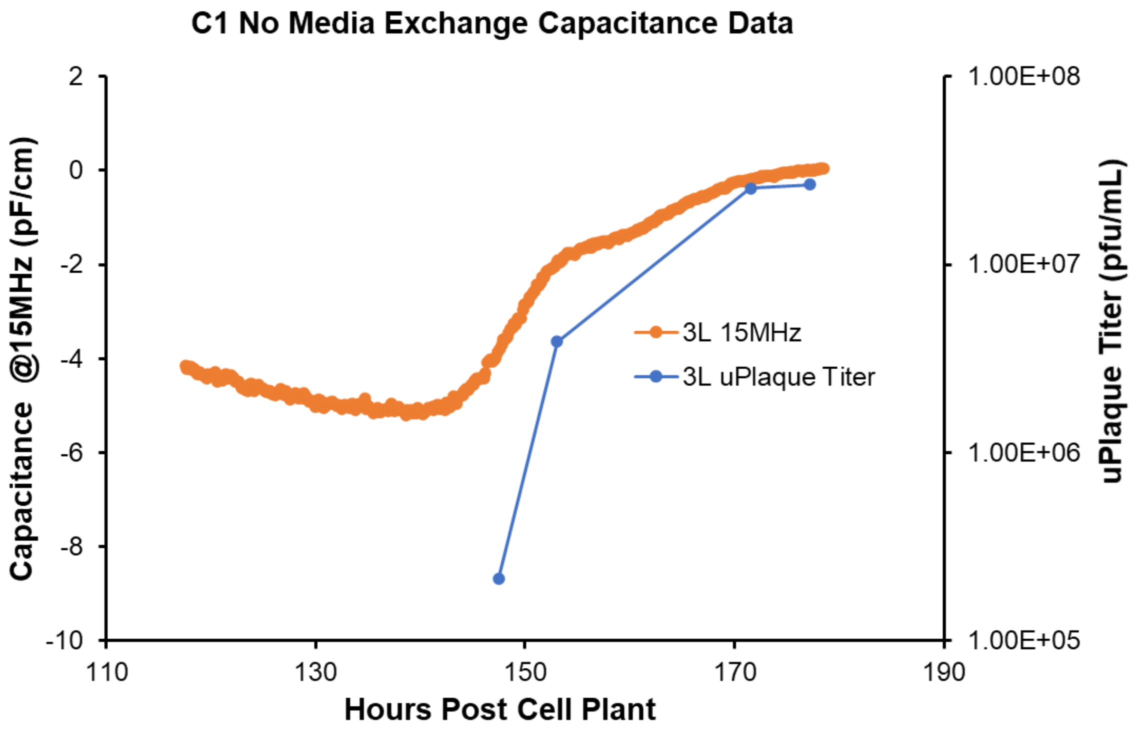

Figure S2. Comparison of 3L C1 vaccinecapacitance data (orange) at high frequency (15MHz) with micro-plaque titer data (blue).

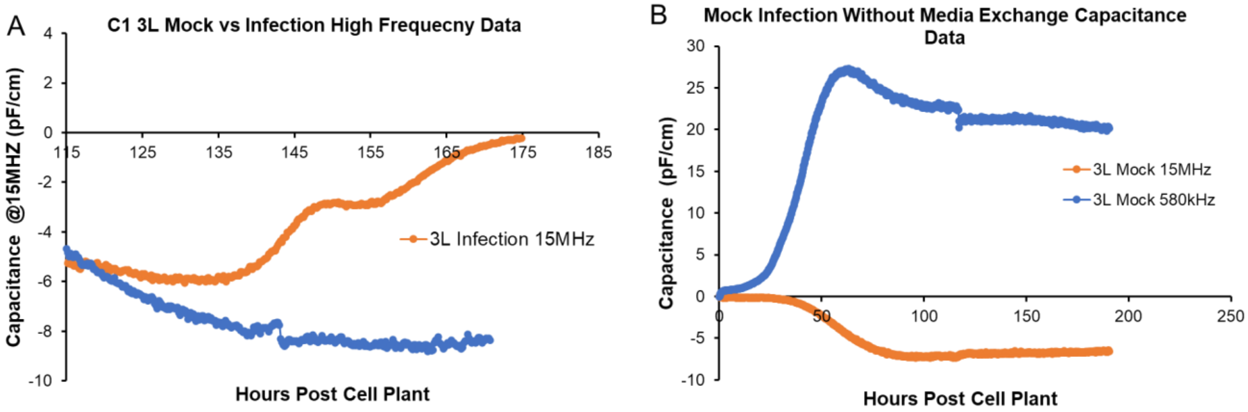

Figure S3. **(A)** Comparison of capacitance profiles at 15MHz for VSV-vectored vaccine (C1) in a 3L bioreactor with a mock infected 3L bioreactor (blue) processed in a similar manner. **(B)** Biomass time profile of mock infected 3L bioreactor without the media exchange unit operation at 580kHz and 15MHz.

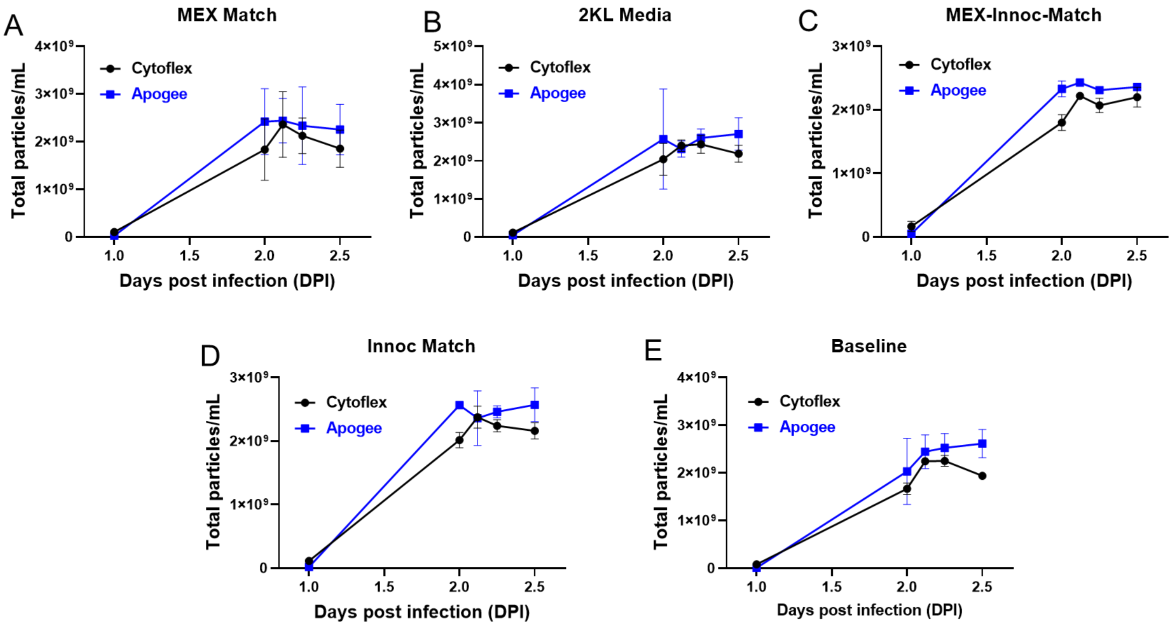

Figure S4. The total particle count of VSV was monitored by two types of flow virometry instruments (CytoFLEX and Apogee) in 3L bioreactors over time under different conditions: 1. MEX Match: separate media formulations where media were formulated in two independent locations **(A)**, 2. 2KL Media: media formulated for 2000L bioreactor and transferred to the 3L reactor **(B)** 3. MEX-Inoculation-Match: media exchange to match cell-adhered microcarrier settling time **(C)** 4. Inoculation Match: cell hold time between n-1 harvest and production bioreactor plant matching 2000L cell transfer time **(D)** and 5. baseline: 3L scale down model control **(E)**.

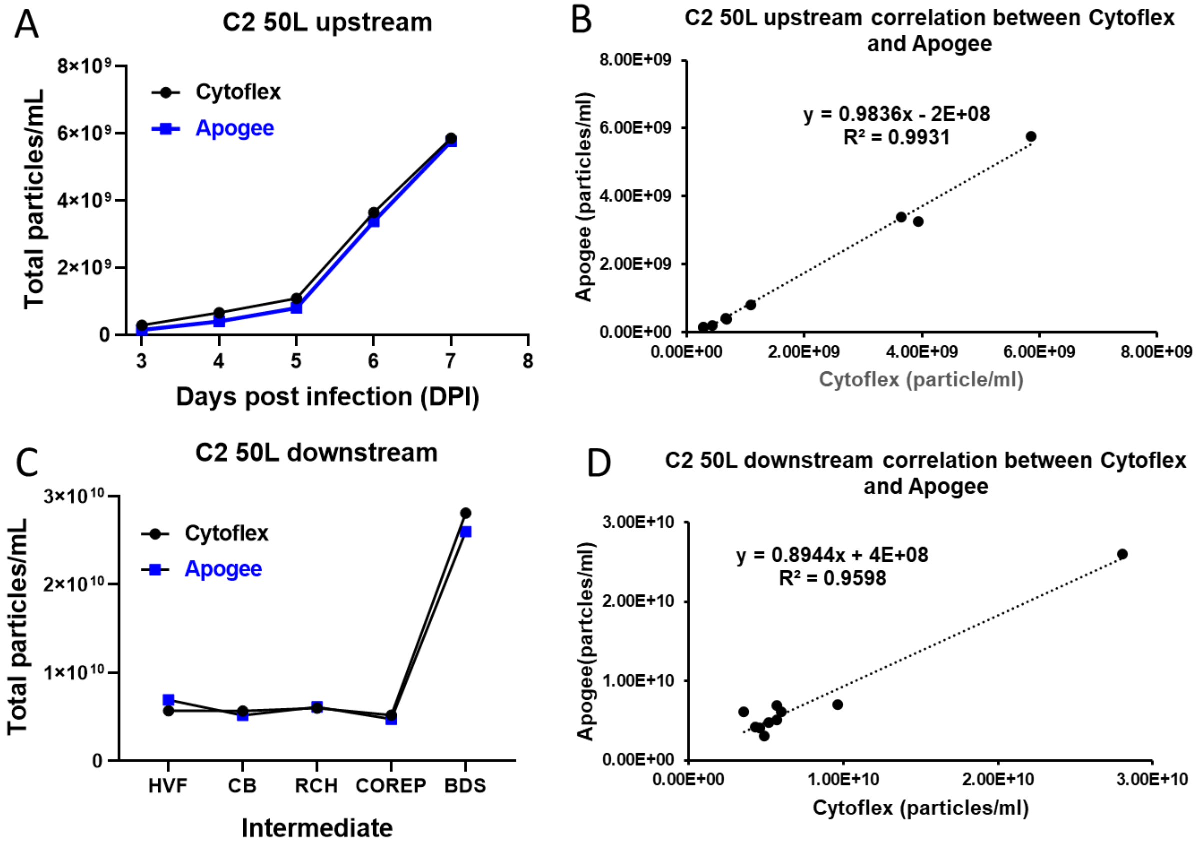

Figure S5. **(A)** The yield of Measles viruses in 50L bioreactors over time was monitored by two types of flow virometry instruments (CytoFLEX and Apogee). **(B)** Correlation between CytoFLEX and Apogee was analyzed for 50L bioreactor samples. **(C)** The yield of Measles viruses in 50L downstream intermediates was measured by both CytoFLEX and Apogee. **(D)** Correlation between CytoFLEX and Apogee was analyzed for 50L downstream samples.
